# Baseline level of interleukin-6 is associated with the risk of acute coronary syndrome development in SARS-CoV‐2 infection

**DOI:** 10.1186/s12872-024-04234-x

**Published:** 2024-10-12

**Authors:** Mohsen Sedighi, Mohammad Hasan Shahabi, Maryam Akbarpour, Alireza Amanollahi, Nader Tavakoli, Aydin Mohammad Valipour, Hamed Basir Ghafouri

**Affiliations:** 1 Trauma and Injury Research Center, Iran University of Medical Sciences, Tehran, Iran; 2 Trauma and Injury Research Center, Rasoul Akram Hospital, Niayesh St, Satarkhan St, Tehran, 14456 Iran

**Keywords:** Acute coronary syndrome, Interleukin-6, Cytokine release syndrome, COVID-19

## Abstract

**Background:**

Acute coronary syndrome (ACS) is frequently reported in patients with coronavirus disease 2019 (COVID-19). Cytokine storm induced by interleukin-6 (IL-6) has been suggested to potentially cause myocardial injury in COVID-19. We investigated the association between baseline level of IL-6 and development of ACS in COVID-19 patients.

**Methods:**

Demographic and clinical data of hospitalized COVID-19 patients from 2020 to 2022 were reviewed. Extracted data including patient characteristics, laboratory biomarkers, and systemic inflammation indexes in patients with or without ACS were reviewed and analyzed. Logistic regression models were applied to analyze predictors of ACS development and receiver-operating characteristic (ROC) curves were used to assess discriminatory power of IL-6 and other risk factors for predicting ACS development.

**Results:**

Among 1,753 COVID-19 patients, 37 cases experienced ACS and 159 patients without main COVID-19 complications were randomly selected as controls. ACS patients were older (*p* = 0.001) and suffered from more comorbidities including diabetes (43% vs. 18%, *p* = 0.001), hypertension (40.5% vs. 24.5%, *p* = 0.050), ischemic heart disease (49% vs. 9%, *p* = 0.001), and hyperlipidemia (19% vs. 5%, *p* = 0.010). Also, decreased level of consciousness (31.6% vs. 2.5%, *p* = 0.001), ICU admission (65% vs. 2%, *p* = 0.001), and mortality events (70% vs. 0.6%, *p* = 0.001) were more prevalent in the ACS group. Baseline levels of IL-6 (*p* = 0.001), D-dimer (*p* = 0.026), troponin (*p* = 0.001), blood urea nitrogen (*p* = 0.002), and creatinine (*p* = 0.008) were higher in ACS patients but erythrocyte sedimentation rate (*p* = 0.013), hemoglobin (*p* = 0.033), and red blood cells (*p* = 0.028) were lower compared with controls. Also, age (OR: 1.06, *p* = 0.019), IL-6 (OR: 1.44, *p* = 0.047), and cardiovascular disease (CVD) (OR: 3.66, *p* = 0.043) were associated with ACS development. The area under the curve (AUC) of IL-6 and combined predictors respectively was 0.661 (*p* = 0.002) and 0.829 (*p* = 0.001).

**Conclusions:**

High IL-6 concentration at baseline is a strong predictor for ACS development in COVID-19 patients. Also, elderly and concurrent CVD are significantly associated with ACS development.

## Introduction

Cardiovascular complications related to coronavirus disease 2019 (COVID-19) such as acute coronary syndrome (ACS), myocardial injury, takotsubo cardiomyopathy, cardiac arrest, and pulmonary thromboembolism have been reported in patients with SARS-CoV‐2 infection (1). These presentations are observed commonly in patients with severe forms of COVID-19 and are associated with poor clinical outcomes (2). Myocardial injury has been found in 20 − 30% of hospitalized COVID-19 patients and may present with electrocardiographic features of ST-segment elevation myocardial infarction (STEMI) (3). The gold standard for diagnosing and treating ACS is restoring coronary blood flow with percutaneous coronary intervention (PCI) but heart failure with reduced ejection fraction (HFrEF) may be observed after PCI (4).

Recent studies have reported that COVID-19 patients are mostly in a high systemic inflammatory condition with excessive cytokine releases which is characterized by high levels of interleukin-6 (IL-6), IL-8, and tumor necrosis factor-α (TNF-α) that contribute to deadly complications (5, 6). Increased level of IL-6 is associated with severe form of COVID-19 and worsening viral disease on the cellular level (7). Furthermore, COVID-19-induced systemic inflammation has been proposed to potentially cause myocardial injury in COVID-19 patients which is initially presented with abnormal C-reactive protein (CRP), N-terminal fragment of pro-BNP (NT-proBNP), and creatinine levels (8). Prior investigation by Moccia et al. in COVID-19 patients showed that non-survivors had a higher level of IL-6 as well as higher rates of acute cardiac injury and acute heart failure, suggesting that IL-6 potentially contributes to development of myocardial injury in COVID-19 patients (9).

In this study, we investigated the association between baseline level of IL-6 and risk of ACS development in a cohort of hospitalized patients with COVID-19 during pandemic.

## Materials and methods

### Study design and participants

This single-center retrospective study was conducted on COVID-19 patients admitted to the one of largest hospitals in our country, exclusively dedicated to the care of COVID-19 patients. Diagnosis of COVID-19 was made based on the World Health Organization (WHO) interim guidance (10) and ACS was diagnosed according to the American Heart Association (AHA) guideline for management of patients with ACS (11).

## Inclusion and exclusion criteria

Inclusion criteria to enter the study were COVID-19 patients who were hospitalized in general or intensive care unit (ICU), aged above 18 years, had available laboratory tests at admission time. Patients were excluded from the study if they left the hospital before the recommendation of physician for discharge or if their medical records were incomplete.

## Data collection

Demographic and clinical data including age and gender, past medical history, typical symptoms and signs of disease, and high-resolution computed tomography (HRCT) of lungs were collected and reviewed from electronic hospital information system (HIS). Also, main laboratory findings on the day of admission including plasma level of IL-6, C-reactive protein (CRP), D-dimer, white blood cell (WBC), monocytes, lymphocytes, neutrophils, and platelets were assessed. Then, we estimated neutrophil-to-lymphocyte ratio (NLR), platelet-to-lymphocyte ratio (PLR), and systemic immune-inflammation index (SII) as indexes of systemic inflammation (platelets × neutrophils/lymphocytes) in the included patients (12).

### Statistical analysis

IBM SPSS version 26.0 (IBM, Inc., Armonk, NY, USA) was used for data analysis. Continuous data were described as mean ± standard deviation (SD) and compared between groups using the Mann-Whitney U test or student t-test based on the normal distribution of data. Categorical variables were represented as frequency (%) and analyzed by Fisher’s exact test. Logistics regression models were applied to determine the association between ACS development and IL-6, age, D-dimer, CRP, and concurrent cardiovascular disease (CVD), as common ACS-related risk factors. Concretely, univariable and multivariable logistic regression models were performed and odds ratio (OR) with 95% confidence interval (CI) was estimated. The ability of predictors to discriminate ACS in COVID-19 patients was determined using a receiver-operating characteristic (ROC) curve based on logistic regression models for IL-6 alone and in combination with other predictors. All p values < 0.05 were considered significant.

## Results

**Fig. 1:**
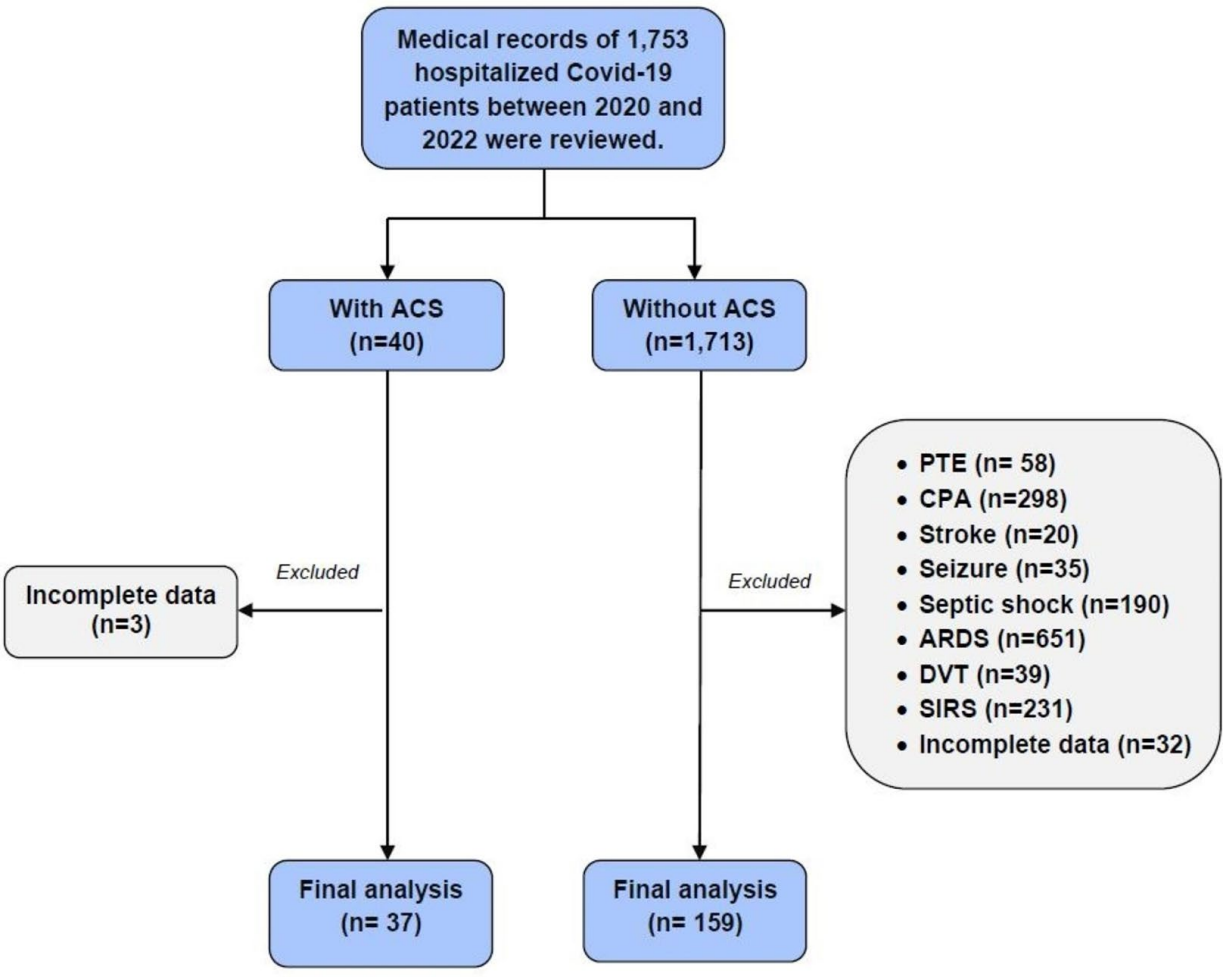
Flowchart of the patient’s population. ARDS, acute respiratory distress syndrome; CPA, cardiopulmonary arrest; DVT, deep vein thrombosis; PTE, pulmonary thromboembolism; SIRS, systemic inflammatory response syndrome

A total of 1,753 hospitalized patients with COVID-19 between 2020 and 2022 were identified and screened. Of these, 40 patients (2.3%) had a confirmed diagnosis of ACS but 3 cases were excluded from the study because of incomplete medical data, and therefore, 37 patients with ACS remained for final analysis. Among other COVID-19 patients, 159 cases who met our inclusion criteria and did not develop main COVID-19 complications such as sepsis, neurological events, systemic inflammatory response syndrome, and thromboembolism were randomly selected for comparison with the ACS group (Fig. 1).

The mean age of total participants was 57.08 ± 15.79 years and 111 (57%) were male. Table 1 compares demographic and clinical characteristics of COVID-19 patients in ACS and control groups. Compared with controls, ACS patients were older (*p* = 0.001) and suffered from more comorbidities including diabetes (*p* = 0.001), hypertension (*p* = 0.050), ischemic heart disease (IHD) (*p* = 0.001), and hyperlipidemia (*p* = 0.010). Also, decreased level of consciousness (*p* = 0.001), ICU admission (*p* = 0.001), and mortality events (*p* = 0.001) were more prevalent in the ACS group. As described in Table 2, a meaningful difference was observed between the groups in red blood cells (*p* = 0.025), hemoglobin (*p* = 0.033), erythrocyte sedimentation rate (*p* = 0.013), blood urea nitrogen (*p* = 0.002) and creatinine (*p* = 0.008). In addition, baseline levels of IL-6 (*p* = 0.001), D-dimer (*p* = 0.026), and troponin (*p* = 0.001) were significantly higher in patients with ACS.

**Table 1:** Characteristics of COVID-19 patients at hospital admission

Variables	ACS group (n = 37)	Control group (n = 159)	P value
Age (years) ^a^	65.38 ± 13.66	55.15 ± 15.67	0.001
Gender (n,%) ^b^ *Male* *Female*	21 (57%) 16 (43%)	90 (57%) 69 (43%)	0.987
Symptoms (n,%) ^b^ *Fever* *Cough* *Dyspnea* *Weakness* *Diarrhea* *Vomit* *Headache* *Musculoskeletal pain*	14 (38%) 24 (65%) 31 (84%) 17 (46%) 1 (3%) 2 (5%) 2 (5%) 14 (38%)	65 (41%) 125 (79%) 130 (82%) 70 (44%) 13 (8%) 16 (10%) 23 (14.5%) 61 (38%)	0.734 0.078 0.828 0.832 0.476 0.534 0.176 0.953
Comorbidities (n,%) ^b^ *DM* *HTN* *IHD* *HLP*	16 (43%) 15 (40.5%) 18 (49%) 7 (19%)	29 (18%) 39 (24.5%) 14 (9%) 8 (5%)	0.001 0.050 0.001 0.010
Systolic BP (mmHg) ^a^	122.43 ± 14.81	119.50 ± 12.35	0.213
Diastolic BP (mmHg) ^a^	77.05 ± 10.37	75.72 ± 10.37	0.604
Heart rate (pulse/min) ^a^	88 ± 16.54	87.17 ± 15.25	0.961
SpO2 (%) ^a^	84.62 ± 7.29	85.18 ± 6.97	0.663
Decreased LOC (n,%) ^b^	6 (31.6%)	2 (2.5%)	0.001
ICU admission (n,%) ^b^	24 (65%)	3 (2%)	0.001
Hospital LOS (days) ^a^	13.35 ± 13.38	10.99 ± 6.86	0.550
Mortality (n,%) ^b^	26 (70%)	1 (0.6%)	0.001

ACS: acute coronary syndrome, BP: blood pressure, COVID-19: coronavirus disease 2019,

DM: diabetes mellitus, HLP: hyperlipidemia, HTN: hypertension, ICU: intensive care unit,

IHD: ischemic heart disease, LOC: level of consciousness, LOS: lengths of stay

^a^ Continues data are presented as mean ± SD

^b^ Categorical data are presented as frequency (%)

**Table 2:** Laboratory indexes of COVID-19 patients at hospital admission

Variables ^a^	ACS group (n = 37)	Control group (n = 159)	P value
WBC (×10^3^/mm^3^)	8.83 ± 4.22	8.73 ± 6.59	0.448
Lymphocyte (%)	17.55 ± 28.29	13.83 ± 8.66	0.554
Neutrophile (%)	85.06 ± 14.52	85.64 ± 10.66	0.431
RBC (million/mm^3^)	4.39 ± 0.67	4.67 ± 0.70	0.028
Hemoglobin (g/dl)	13.04 ± 1.98	13.80 ± 1.94	0.033
Hematocrit (%)	38.39 ± 5.63	40.16 ± 5.02	0.060
Platelet (×10^3^/mm^3^)	191.35 ± 64.88	196.49 ± 71.47	0.689
NLR	9.35 ± 6.51	10.07 ± 10.50	0.586
PLR	244.72 ± 159.50	272.58 ± 272.24	0.864
SII (*10 ^− 3^)	1.83 ± 1.41	2.16 ± 3.04	0.739
CRP (mg/L)	22.31 ± 9.54	21.53 ± 6.20	0.561
ESR (≤ 15 mm/h)	37.29 ± 28.05	47.31 ± 26.96	0.013
P-LCR (17–45%)	25.46 ± 7.78	23.73 ± 12.12	0.644
IL-6 (1-16.4 pq/ml)	348.03 ± 633.39	111.03 ± 204.81	0.001
D-dimer (ng/mL*)*	808.777 ± 990.70	496.91 ± 1310.32	0.026
PT (9–13 s)	14.78 ± 2.02	14.35 ± 2.81	0.102
PTT (25–35 s)	35.46 ± 8.13	33.73 ± 7.03	0.203
INR (0.9–1.2)	1.15 ± 0.19	1.34 ± 2.66	0.462
BUN (14–45 mg/dl)	24.19 ± 17.97	18.43 ± 7.18	0.002
Creatinine (0.7–1.4 mg/dl)	1.50 ± 1.17	1.21 ± 0.35	0.008
Sodium (135–145 mEq/L)	136.38 ± 5.23	136.78 ± 3.47	0.659
Potassium (3.5–5.5 mEq/L)	4.41 ± 0.64	4.26 ± 0.57	0.181
ALP (30–130 U/L)	214.82 ± 101.16	192.74 ± 89.75	0.211
CPK (24–195 U/L)	405.05 ± 738.78	320.55 ± 496.19	0.594
LDH (225–500 U/L)	913.91 ± 538.26	780.13 ± 307.15	0.170
AST (≤ 40 U/L)	82.86 ± 66.03	75.77 ± 50.21	0.663
ALT (≤ 40 U/L)	70.72 ± 62.88	65.29 ± 37.09	0.666
Troponin	251.06 ± 193.63	15.33 ± 36.02	0.001

ACS: acute coronary syndrome, ALP: alkaline phosphatase, ALT: alanine transaminase, AST: aspartate aminotransferase, BUN: blood urea nitrogen, COVID-19: coronavirus disease 2019, CPK: creatine phosphokinase, CRP: C-reactive protein, ESR: erythrocyte sedimentation rate, IL-6: interleukin-6, INR: international normalized ratio, SII: systemic immune-inflammation index, LDH: lactate dehydrogenase, NLR: neutrophil to lymphocyte ratio, P-LCR: platelet-large cell ratio, PLR: platelet to lymphocyte ratio, PT: prothrombin time, PTT: partial prothrombin time, RBC: red blood cell, WBC: white blood cell. ^a^ Continues data are presented as mean ± SD

**Fig. 2:**
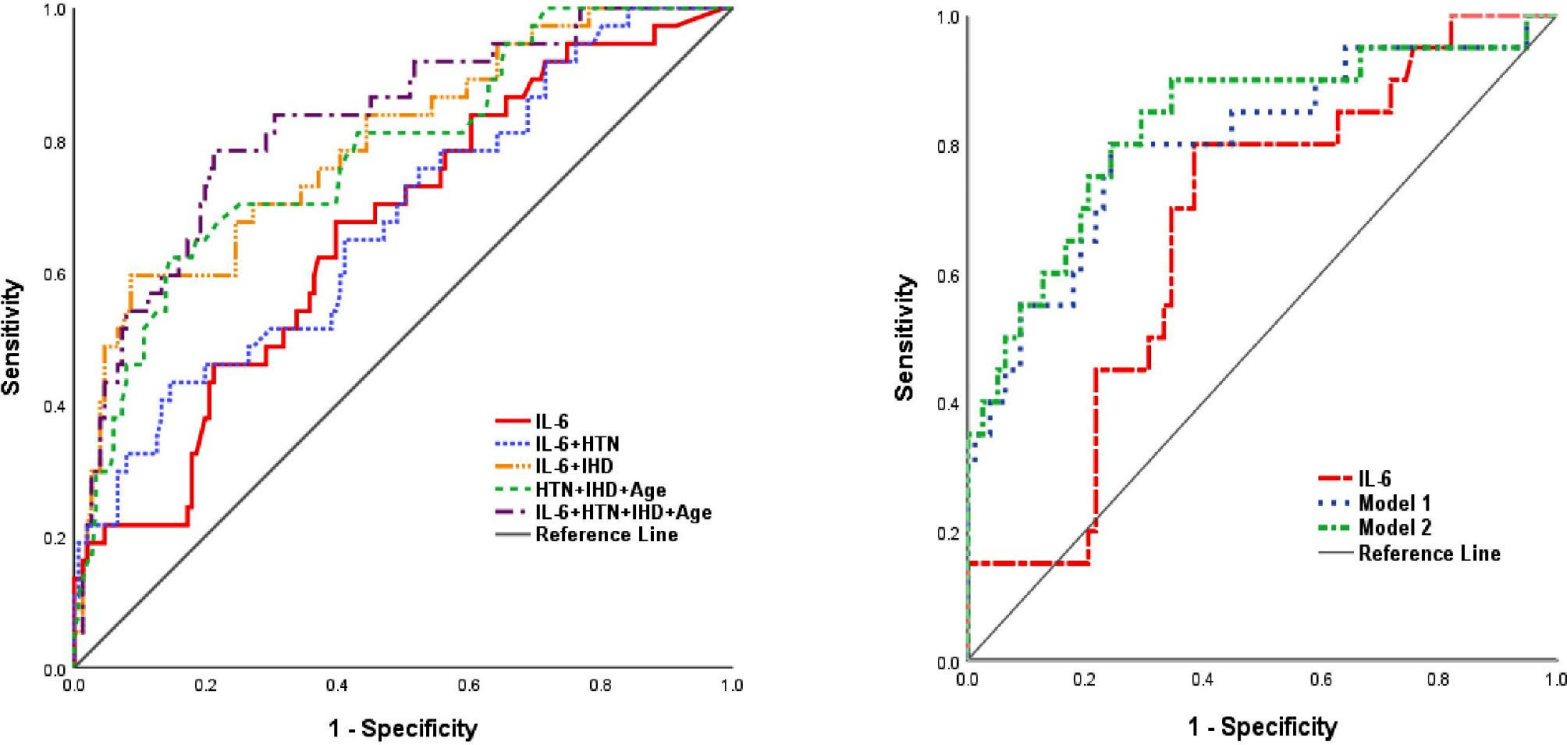
ROC curve of IL-6 and other predictors to discriminate development of ACS in COVID-19 patients. Model 1 = CVD + CRP + D-dimer + creatinine + age, Model 2 = IL-6 + age + CVD + CRP + D-dimer + creatinine

The results of univariable and multivariable logistic regression models to evaluate the association between variables of COVID-19 patients and ACS development are shown in Table 3. In univariable analysis, age (OR = 1.04), IL-6 (OR = 2.66), creatinine (OR = 1.94), and CVD (OR = 3.83) were associated with ACS. The result of multivariable models showed that IL-6 (OR = 1.44), age (OR = 1.06), and CVD (OR = 3.66) were correlated with ACS. To determine the prognostic ability of IL-6 and other predictors for ACS development, ROC curves were generated. Accordingly, IL-6 could discriminate ACS by AUC of 0.661 (95% CI = 0.565–0.757; *p* = 0.002). In addition, inflammatory indexes, age, and clinical factors were combined into multivariable models. Model 2 (IL-6 + age + CVD + CRP + creatinine + D-dimer) showed the highest AUC value (AUC = 0.829 [95% CI = 0.716–0.942], *p* = 0.001) for predicting ACS. The AUC values are shown in Table 4; Fig. 2.

**Table 3:** Logistics regression for the association of patient variables with ACS development

Variables		Univariable			Multivariable	
		OR (95% CI)	P value		OR (95% CI)	P value
Age		1.04 (1.02–1.07)	0.001		1.06 (1.01–1.11)	0.019
IL-6		2.66 (1.14–6.21)	0.023		1.44 (1.00–2.08)	0.047
CRP		0.83 (0.47–1.47)	0.533		2.69 (0.45–15.97)	0.274
D-dimer		1.11 (0.96–1.28)	0.146		1.12 (0.95–1.33)	0.169
Creatinine		1.94 (1.02–3.68)	0.041		0.38 (0.06–2.28)	0.293
CVD		3.83 (1.82–8.05)	0.001		3.66 (1.03–12.92)	0.043

ACS: acute coronary syndrome, CI: confidence interval, CRP: C-reactive protein, CVD: cardiovascular disease, IL-6: interleukin-6, OR: odds ratio

**Table 4:** ROC analysis for discriminate ACS development in COVID-19 patients

Predicted indicator	AUC of ROC	95% CI		P value
IL-6	0.661	0.565–0.757		0.002
IL-6 + HTN	0.675	0.578–0.773		0.001
IL-6 + IHD	0.792	0.709–0.875		0.001
HTN + IHD + age	0.779	0.695–0.864		0.001
IL-6 + HTN + IHD + age	0.825	0.749–0.900		0.001
Model 1*	0.801	0.681–0.921		0.001
Model 2**	0.829	0.716–0.942		0.001

Model 1* = CVD + CRP + D-dimer + creatinine + age

Model 2** = IL-6 + age + CVD + CRP + D-dimer + creatinine

ACS: acute coronary syndrome, AUC: area under curve, CI: confidence interval, COVID-19: coronavirus disease 2019, CVD: cardiovascular disease, HTN: hypertension, IHD: ischemic heart disease, IL-6: interleukin-6, ROC: receiver-operating characteristic

## Discussion

ACS is defined by a spectrum of acute myocardial ischemia due to the sudden decline in coronary blood flow, ranging from unstable angina to myocardial infarction (13). ACS has been reported frequently in COVID-19 patients and the risk of ACS is 13-fold higher in individuals with severe clinical presentation (14, 15). In the present study, we evaluated the development of ACS and related risk factors in a cohort of hospitalized COVID-19 patients, with a special focus on the assessment of inflammatory indexes. ACS was observed in 2.3% of patients in our cohort, of which 65% were hospitalized in ICU. In this study, we showed that high level of baseline IL-6 (OR = 2.6) and concurrent CVD (OR = 3.8) were associated with ACS development in SARS-CoV‐2 infection.

Several potential mechanisms for the pathogenesis of ACS in COVID-19 have been proposed but systemic inflammatory response with cytokine-mediated injury and microvascular thrombosis are the most important determinants (16). The clinical course of severe forms of COVID-19 is typified by an aberrant inflammatory response and cytokine storm. SARS-CoV-2-induced cytokine storm is characterized by increased levels of proinflammatory factors including IL-6, IL-10, and TNF-α (17). In response to this inflammatory state, platelet tissue factor (TF) is released from the monocyte-derived macrophages and endothelial cells. TF affects the coagulopathy and stimulates the external coagulation pathway due to fibrin degradation product (D-dimer) and blood clotting pathway activity (18). Elevated levels of D-dimer, CRP, fibrinogen, von Willebrand factor (VWF), ferritin, complement, and IL-6 affect both arterial and venous circulation that increases risk of developing thrombotic events, resulting in microvascular thrombosis (19).

Large amounts of IL-6 have been found in human atherosclerotic plaque, in particular within the shoulder region of stable and unstable plaque (20). In addition, IL-6 level in patients with ACS is shown to be markedly higher at the site of coronary plaque rupture than in the systemic circulation (21). Viral infections can increase sympathetic activity with consequent vasoconstriction in the coronary arteries. Therefore, the interplay between all these biological and mechanical conditions can induce atheromatous plaque erosion or rupture, leading to coronary thrombosis and ACS in COVID-19 patients (22). The 2.6-fold increase in baseline level of IL-6 in ACS patients in our study supports prior investigations and the hypothesis that cytokine storm induced by IL-6 disrupts physiological homeostasis that results in thrombotic events and coronary tissue injury (8).

Concurrent comorbidities like CVD are potential risk factors for the severe form of COVID-19 and cause a range of cardiac complications, including myocardial injury, myocarditis, arrhythmias, cardiomyopathy, heart failure, and thromboembolic events (22). In our study, old age in COVID-19 patients was meaningfully associated with ACS development (OR = 1.04) and also patients with concurrent CVD had nearly 3.8 times the risks for ACS development. CVD is a marker of accelerated immunologic aging and immune dysregulation that influences prognosis of patients indirectly. Atherogenic index of plasma (AIP) is found to be an important marker affecting pre-PCI thrombolysis in myocardial infarction (TIMI) flow (23). Furthermore, patients with hypertension and CVD might have increased expression of angiotensin-converting enzyme-2 (ACE-2) receptors that increase patients’ susceptibility to SARS-CoV‐2 infection (24). Recent investigation has shown that hypertensive patients have a hypercoagulability status due to higher neutrophil extracellular traps (NETs) formation which is correlated with underlying inflammatory reactions and may exacerbate endothelial injury and COVID-19 severity (25).

The current study has some potential limitations that should be noted. This was a single-center and retrospective study that was performed on hospitalized COVID-19 patients, whereas mild or asymptomatic COVID-19 patients were excluded. Also, the small sample size of ACS patients is a major limitation that can affect the results of our study. Thus, more studies are warranted to gain a better understanding of the risk factors for ACS associated with COVID-19. Furthermore, some laboratory indexes were not performed in all patients, and missing data might lead to bias in the analysis of patient characteristics.

## Conclusions

The results of present study showed that high level of IL-6 at baseline is a strong predictor for ACS development in COVID-19 patients. In addition, elderly and concurrent CVD are significantly associated with ACS in patients with SARS-CoV‐2 infection.

## Data Availability

The data that supports the findings of this study are available from the corresponding author upon reasonable request.

## Abbreviations

ACS: Acute coronary syndromes
COVID-19: Coronavirus disease 2019
CRP: C-reactive protein
CVD: Cardiovascular disease
DM: Diabetes mellitus
HLP: Hyperlipidemia HTN: Hypertension
IHD: Ischemic heart disease
IL-6: Interleukin-6
NLR: Neutrophil to lymphocyte ratio
PLR: Platelet to lymphocyte ratio
SII: Systemic immune-inflammation index
HRCT: High-resolution computed tomography
HIS: Hospital information system
ROC: Receiver-operating characteristic
WBC: White blood cell
